# Supercritical Carbon Dioxide Extraction of Astaxanthin, Lutein, and Fatty Acids from *Haematococcus pluvialis* Microalgae

**DOI:** 10.3390/md16090334

**Published:** 2018-09-13

**Authors:** Giuseppe Di Sanzo, Sanjeet Mehariya, Maria Martino, Vincenzo Larocca, Patrizia Casella, Simeone Chianese, Dino Musmarra, Roberto Balducchi, Antonio Molino

**Affiliations:** 1 ENEA, Italian National Agency for New Technologies, Energy and Sustainable Economic Development, Department of Sustainability, CR Trisaia SS Jonica 106, km 419+500, 75026 Rotondella, Italy; giuseppe.disanzo@enea.it (G.D.S.); maria.martino@enea.it (M.M.); vincenzo.larocca@enea.it (V.L.); roberto.baldicchi@enea.it (R.B.); 2 ENEA, Italian National Agency for New Technologies, Energy and Sustainable Economic Development, Department of Sustainability, CR Portici. P. Enrico Fermi, 1, 80055 Portici, Italy; smehariya@gmail.com (S.M.); patrizia.casella@enea.it (P.C.); 3 Department of Engineering, Università degli Studi della Campania “L. Vanvitelli”, Real Casa dell’Annunziata, Via Roma 29, 81031 Aversa, Italy; sim.chianese@gmail.com (S.C.); dinomusmarra@gmail.com (D.M.)

**Keywords:** microalgae, *Haematococcus pluvialis*, astaxanthin, lutein, fatty acids, supercritical fluid extraction, natural medicines

## Abstract

*Haematococcus pluvialis* microalgae in the red phase can produce significant amounts of astaxanthin, lutein, and fatty acids (FAs), which are valuable antioxidants in nutraceutics and cosmetics. Extraction of astaxanthin, lutein, and FAs from disrupted biomass of the *H. pluvialis* red phase using carbon dioxide (CO_2_) in supercritical fluid extraction (SFE) conditions was investigated using a bench-scale reactor in a semi-batch configuration. In particular, the effect of extraction time (20, 40, 60, 80, and 120 min), CO_2_ flow rate (3.62 and 14.48 g/min) temperature (50, 65, and 80 °C), and pressure (100, 400, and 550 bar.) was explored. The results show the maximum recovery of astaxanthin and lutein achieved were 98.6% and 52.3%, respectively, at 50 °C and 550 bars, while the maximum recovery of FAs attained was 93.2% at 65 °C and 550 bars.

## 1. Introduction

In the last few years, microalgae have attracted growing attention for producing a wide range of “high value-added compounds”, that are useful in several aspects of our life. Literature data show that microalgae are composed of molecules that can be used in many industrial sectors such as pharmaceutics, nutraceutics, food additives, and natural medicines, along with natural cosmetics [1,2,3,4,5]. These markets are characterized by high-quality safe-products at competitive prices. In this context, microalgae represent the starting point for the production of natural compounds with a sustainable approach characterized by low environmental impact [6,7]. Microalgae grow in diverse environments as freshwater/sea water and use sun light and carbon dioxide for photosynthesis [7,8]. They can be grown in photobioreactors or open ponds that can be located in marginal or unproductive land, and without the use of herbicides or pesticides, thus allowing for the reduction of the environmental impact related to cultivation system [9]. Another advantage of microalgae is that they can be used for carbon dioxide sequestration in their growth phase; in fact, each kilogram of dry biomass of microalgae could capture about 1.8–2 kg of CO_2_. As a consequence, microalgae can be used to reduce CO_2_ emission [1,10,11,12,13].

Astaxanthin, lutein, and fatty acids (FAs) naturally accumulate in several marine species including the microalgae *H. pluvialis,* which contains the highest levels per cell. Due to the strong antioxidant and antiaging properties from *H. pluvialis*, it has been cultivated by several industries [4,13,14,15,16]. *H. pluvialis* are unicellular representatives of the phylum Chlorophyta, which can be found in freshwater, marine, or even terrestrial environments [17]. Chekanov et al. [18] isolated *H. pluvialis* strain BM1 from the White Sea Coastal Rocks (66°29′47″ N, 33°24′22″ E) (Russia), which can accumulate a significant amount of astaxanthin under unfavourable environmental conditions. Moreover, the effect of salinity was analyzed on astaxanthin production in *H. pluvialis* growth [19]. Therefore, microalgae *H. pluvialis* could be found marine environments. However, the accumulation of these “high value-added products” within *H. pluvialis* cells is related to the hard cell wall that is highly resistant to both chemical and physical disruptions [20,21,22]. Unfortunately, the extraction of these compounds from *H. pluvialis* red microalgae is a major obstacle to recover these compounds with high purity in a cost-effective and eco-friendly manner. Therefore, the choice of a suitable extraction method depends on several aspects such as biomass, as well as extract, its end use, and its thermolability [23,24,25].

Conventional extraction techniques used for vegetables include squeezing, maceration, infusion, percolation, steam distillation, and solvent extraction. These techniques often have several issues related to the thermal degradation, which is due to their high extraction temperature; the solvent residues in the extracts can also compromise their end uses [24,26,27,28]. Moreover, in the last decade the scientific community has proposed advanced extraction techniques such as ultrasonic extraction, microwave extraction, accelerated solvent extraction, and extraction with supercritical fluid (SFE) to reduce these issues and minimize energy costs and the environmental impact [29,30,31,32]. Supercritical fluid extraction using CO_2_ as an extraction fluid in supercritical condition (SFE-CO_2_) represents a valid alternative to conventional techniques when it is necessary to guarantee thermal stability, and high-quality products (terms of purity and yield without solvent traces) [33]. Also, SFE-CO_2_ is an eco-friendly technique for the obtaining of “high value-added compounds” from different matrices [14,28]. Moreover, CO_2_ is not reactive at low temperatures, and it is easily recovered after each extraction stage [34]. Therefore, SFE-CO_2_ is a novel technology with great potential for the extraction of bioactive compounds, which are used as food additives in the nutraceutical field [27,35,36,37]. Literature shows that several experimental tests have been carried out on the extraction of astaxantin from *H. pluvialis* using SFE-CO_2_ [14,38,39,40]. Lutein extractions using SFE-CO_2_ from other microalgal species, such as *Chlorella* [11,24,41] and *Scenedesmus* [9,42,43,44,45], have been investigated.

In this paper, the extraction performance of astaxanthin, lutein, and FAs from *H. pluvialis* in the red phase was assessed, using bench-scale SFE-CO_2_ installation. In order to promote the extraction of astaxanthin, lutein, and FA, the cell wall of *H. pluvialis* red biomass was disrupted by mechanical (ball milling) pre-treatment [14], as optimized in our previous study [3]. The effect of different operative conditions, i.e., CO_2_ flow rate (3.62 g/min and 14.48 g/min), run time (20–120 min), extraction temperature (50–80 °C), and pressure (100–550 bars) on the recovery and purity of astaxanthin, lutein, and FAs, was investigated, in order to find the best conditions to obtain the highest recovery and purity of all the compounds considered. Moreover, the characterization of the FAs extracted was performed, in terms of saturated fatty acids (SFAs), monounsaturated fatty acids (MFAs), and polyunsaturated fatty acids (PUFAs), in order to evaluate the effectiveness of the SFE-CO_2_ extraction technique for the recovery of FAs species.

## 2. Materials and Methods

### 2.1. H. pluvialis Red Biomass and Chemicals

*H. pluvialis* in red phase (HPR) in powder was obtained by Micoperi Blue Growth^®^, an Italian company. HPR presented a mesh particle sieve lower than 50 μm, which contains 20 mg astaxanthin/g_dry biomass_, 7.7 mg lutein/g_dry biomass_, and 22.96 mg FAs/g_dry biomass_. The lipid content was 2.6% by wt/wt of biomass, while the FAs content was 88.3% of total lipids. The saturated fatty acids (SFAs), monounsaturated fatty acids (MFAs), and polyunsaturated fatty acids (PUFAs) were 28.1%, 23.7%, and 48.2% of total FAs. Also, the chemical characterization of biomass was carried out using standard methods and summarized in our earlier study [3]. The biomass was stored at −20 °C and brought to room temperature before use. Carbon dioxide (Industrial grade) was obtained from Rivoira, Italy; astaxanthin, lutein, and FAs (analytical grade) were purchased from Sigma Aldrich, St. Louis, MO, USA. All other reagents were uHPLC grade unless otherwise stated.

### 2.2. Experimental Apparatus

**Figure 1:**
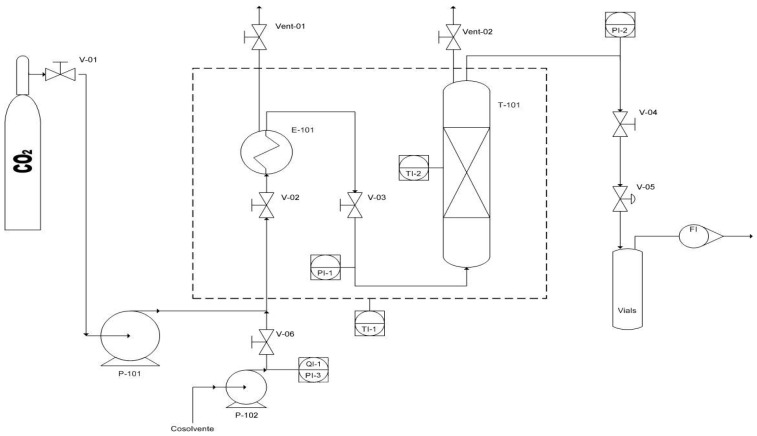
Schematization of the bench scale SFE-CO_2_ experimental apparatus.

Experimental tests were carried out using mechanically pre-treated dry microalgae and using the bench scale extractor SPE-ED SFE 2 by Applied Separations (Figure 1), which is characterized by a reactor volume of 30 mL. The mechanical pre-treatment of HPR biomass was carried out using a ball mill at 400 rpm for 5 min as described by Molino et al. [3]. The bench scale SFE-CO_2_ is equipped with a heater able to achieve temperature up to 250 °C and a pumping system for the compression of carbon dioxide up to 680 bar. Two vessels are located inside the module: The first is used as CO_2_ pre-heater, and the second one is the vessel where the extraction was carried out. In the extraction vessel there are two pressure controllers (Inlet and Outlet valves) and a Wika Transmitter with a precision of 0.6 mbar, whereas the CO_2_ flow rate is monitored by using flow meter LPN/S80 AL G 2.5 by Sacofgas. The inlet flow rate is adjustable to 25 mL/min, and the flow control is done on the expanded gas. All parameters are controlled with a Distributed Control System (DCS). The temperature is monitored by thermocouples, while inlet and outlet flow are controlled by using micrometric valves.

**Figure 2:**
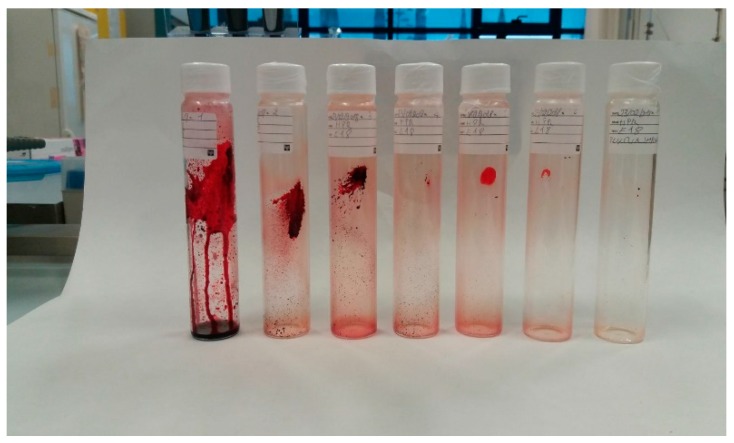
Extract from the bench scale SFE-CO_2_ extractor.

A picture of the extract is shown in Figure 2.

### 2.3. Experimental Procedure

Before the extraction processes, to achieve uniform cell disruption and maximum product recovery [14] and to promote the extraction of astaxanthin, lutein, and FA, the cell wall of *H. pluvialis* red (HPR) biomass was disrupted by mechanical (ball milling) pre-treatment, as optimized in our pervious study [3]. In each run, around 1.4 g of biomass was mixed with 0.8 g of diatomaceous earth, which was disrupted and homogenized as described in earlier studies [3]. This quantity of mixture biomass/diatomaceous earth was loaded into the extraction cell, and free volume was filled by using diatomaceous earth till to complete the internal volume of the cell.

The disrupted cells were then extracted using SFE-CO_2_ at different operative conditions. In particular, the effect of CO_2_ flow rate (3.62 g/min and 14.48 g/min), time (20–120 min, extraction stage = 20 min), temperature (50–80 °C), and pressure (100–550 bar) were tested. These SFE-CO_2_ parameters significantly influence the extraction efficiency, as well as selectivity of target compounds for extraction [14]. Therefore, these parameters have to be carefully considered and optimized for an efficient and selective recovery of target products. The experimental conditions are summarized in Table 1.

The effect of operative conditions on astaxanthin, lutein, and FAs extraction was expressed in terms of recovery and purity, which were calculated on the basis of initial weight of each compound in HPR [3] as reported in the following:

*recovery* (%) = *W_c_*/*W_t_* × 100 (1)

(2) $$purity\;\left(\%\right)=W_{C}/W_{E}\times 100\;$$

in which *W_C_* is the weight of the compound extracted (mg); *W_T_* is the theoretical weight of the compound from conventional extraction (mg), which was calculated on the basis of the initial content of each compound in HPR, expressed as mg of extract per gram of dry weight of *H. pluvialis*, equal to = 20 mg/g for astaxanthin and equal to 7.7 mg/g for lutein; and *W_E_* is the weight of the extract (mg).

Each experimental condition was replicated three to five times, and for each value standard deviation (SD) was calculated.

The extracted product was dissolved in acetone and stored at −30 °C with the exclusion of light prior to subsequent analysis.

### 2.4. Analytical Methods

The amount of astaxanthin and lutein in the extracts was measured by u-HPLC (Agilent 1290 Infinity II with Agilent Zorbax Eclipse plus C18 column 1.8 μm) [3]. The u-HPLC was equipped with a quaternary pump, thermostated oven column, and UV diode array detector (DAD) (measuring absorbance at 444–450–478 nm). A mixture of methanol/water (95:5%) was used as the mobile phase solvent in isocratic flow, while the sample was dissolved in a mixture of methanol/chloroform (90:10 containing 0.1% BHT as antioxidant agent). The flow rate and column temperature were kept constant at 0.4 mL/min and 28 °C, respectively. Gas chromatograph was used for the analysis of FAs, which was equipped with Flame Ionization detector (FID), a column HP-88 100 mt × 0.25 mm × 0.2 μm. This chromatographic column produced by Agilent is composed of a high polarity bis (Cyanopropyl) siloxane stationary phase and was chosen for its high resolution of positional and geometric isomers of fatty acid methyl esters. The column was maintained at 150 °C for 5 min and was followed by temperature ramping at 1.6 °C/min to 180 °C, then at 1.4 °C/min to 190 °C, and finally holding the temperature at 190 °C for 10 min. Nitrogen (purity ≥ 99.9999%) was used as carrier gas with a linear velocity of 30 cm/s and split ratio of 1:100. The injection port and detector were maintained at 250 °C. To quantify the concentration of the astaxanthin, lutein, and FAs compounds, the calibration curves were built by using chromatographic standards bought by Sigma Chemical Co., St Louis, MO, USA.

## 3. Result and Discussion

Total extraction yields for each operative condition are summarized in Table 1. The results are expressed as mg of extract per g dry weight of HPR, and values were obtained at the end of extraction (120 min, sum of each stage). At a pressure of 100 bars was measured the lowest exaction yields, which are among the values reported in Table 2; therefore, astaxanthin and lutein content at 100 bars for each stage were not analyzed.

### 3.1. Effect of Different CO_2_ Flow Rate at Different Temperatures and Pressure on Astaxanthin Recovery and Purity over Extraction Time

**Figure 3:**
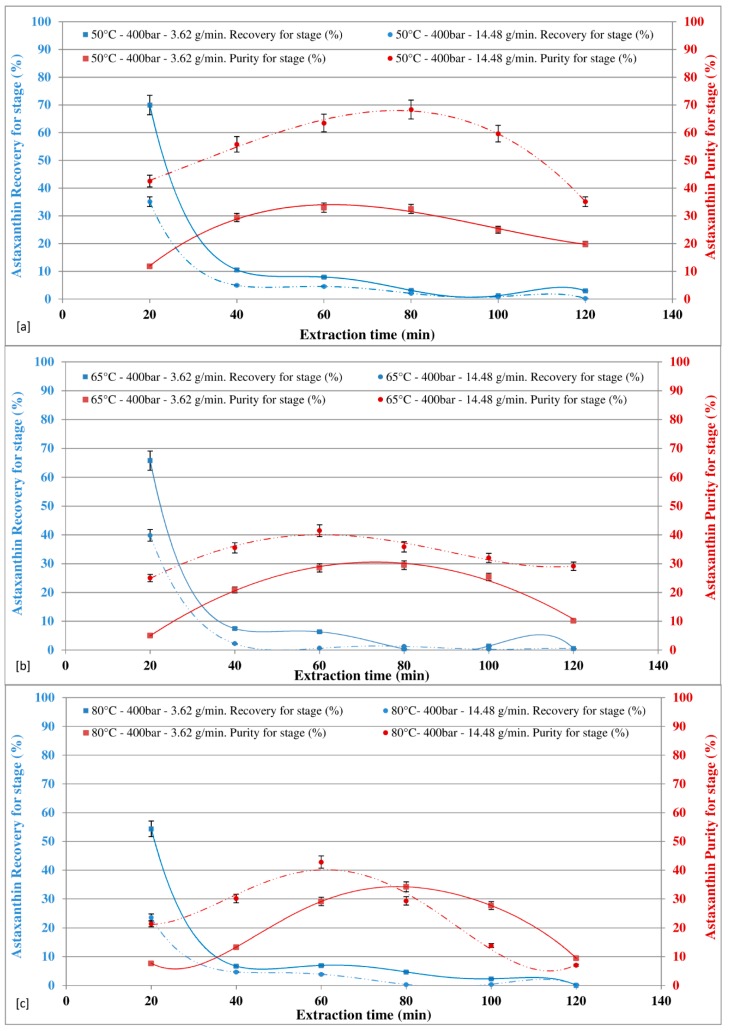
Effect of CO_2_ flow rate at different temperatures on astaxanthin recovery and purity in extract at 400 bar.

**Figure 4:**
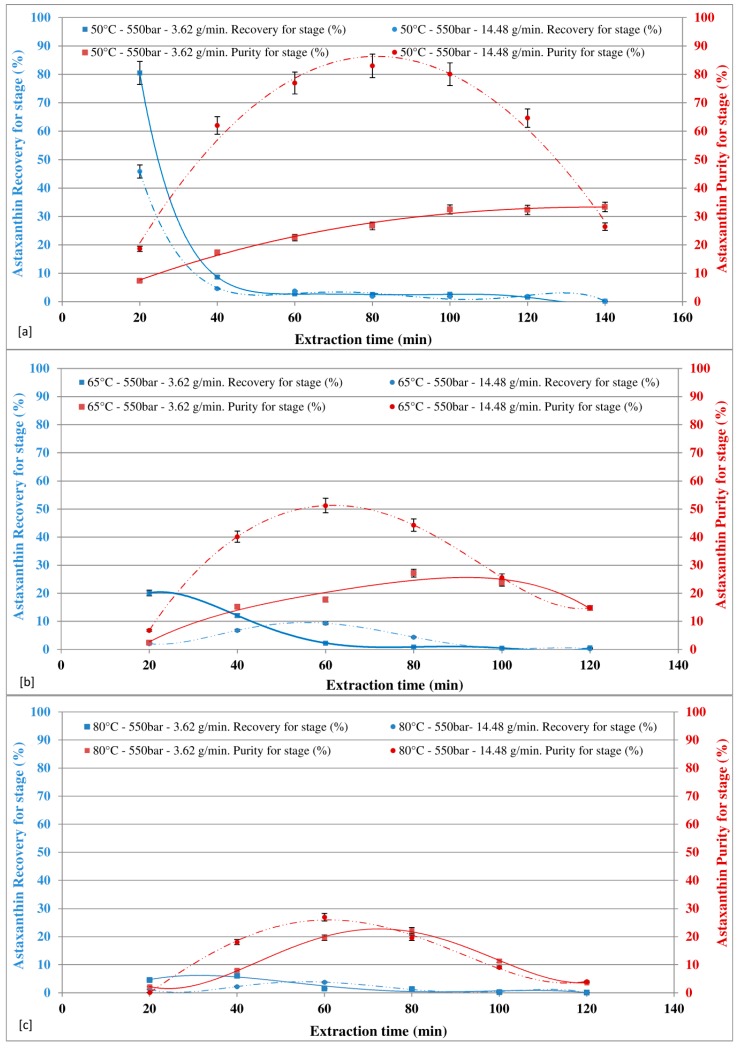
Effect of CO_2_ flow rate at different temperature on astaxanthin recovery and purity in extract at 550 bar.

The effects of CO_2_ flow rate (3.62 g/min and 14.48 g/min), as a function of the extraction time, on SFE-CO_2_ astaxanthin extraction were investigated by operating the temperature of the reaction chamber to 50 °C, 65 °C, and 80 °C and by keeping the pressure at 400 bar (Figure 3a–c) and at 550 bars (Figure 4a–c).

At 400 bars (Figure 3a–c), both the astaxanthin recovery and the astaxanthin purity were affected by the CO_2_ flow rate. In particular, along with extraction time, for all the temperatures investigated, the lower the CO_2_ flow rate, the higher the recovery, and for 50 °C and 65 °C, the higher CO_2_ flow rate, the higher the purity. Around 70% astaxanthin recovery was achieved in first extraction cycle (20 min) with a CO_2_ flow rate of 3.62 g/min and 35% at CO_2_ flow rate of 14.48 g/min. A maximum purity of 68% astaxanthin was achieved at 50 °C with a CO_2_ flow rate of 14.48 g/min and an extraction time of 80 min, while at the same temperature and pressure, the maximum amount of the total extract was achieved with a CO_2_ flow rate of 3.62 g/min (136.4 mg/g).

At 550 bars (Figure 4a–c), for all the temperatures investigated, the astaxanthin recovery was affected by the CO_2_ flow rate; for the first extraction cycle and the second extraction cycle (40 min), in particular, the lower the CO_2_ flow rate, the higher the recovery. Around 90% astaxanthin recovery was achieved in first and second extraction cycles (40 min), with a CO_2_ flow rate of 3.62 g/min and about 35% at CO_2_ flow rate of 14.48 g/min. In terms of astaxanthin purity, the higher the CO_2_ flow rate, the higher the purity. The maximum purity of astaxanthin of about 83% was achieved at 50 °C with a CO_2_ flow rate of 14.48 g/min and an extraction time of 80 min, while at the same temperature and pressure, the maximum amount of the total extract was achieved with a CO_2_ flow rate of 3.62 g/min (237.4 mg/g).

Machmudah et al. [46] suggested that the total extract slightly increased with increasing CO_2_ flow rate, while the amount of astaxanthin recovery and the astaxanthin content in the extract almost did not change. Literature data also shows that the flow rate from 0.9 g/min to 1.8 g/min could not show clear influence on astaxanthin recovery and tended toward the same value at higher CO_2_ consumption. In our study, investigations were carried out by using approximately 1.4 g of HPR. Furthermore, the clear effect of CO_2_ flow rate were observed on astaxanthin recovery, as well as purity, due the range of tested flow rates (i.e., 3.62 and 14.48 g/min). A greater flow rate could be used to achieve the maximum purity, while a smaller flow rate could be used to achieve maximum recovery of astaxanthin from *H. pluvialis* red (HPR) biomass. Therefore, SFE-CO_2_ with greater flow rate has the potential to be directly use in food products, avoiding the need for the solvent separation and purification [47].

By increasing temperature from 50 °C to 80 °C, both at 400 bars and at 550 bars, the recovery and purity decrease.

### 3.2. Effect of Different CO_2_ Flow Rate with Different Temperatures on Lutein Recovery and Purity over Extraction Time

**Figure 5:**
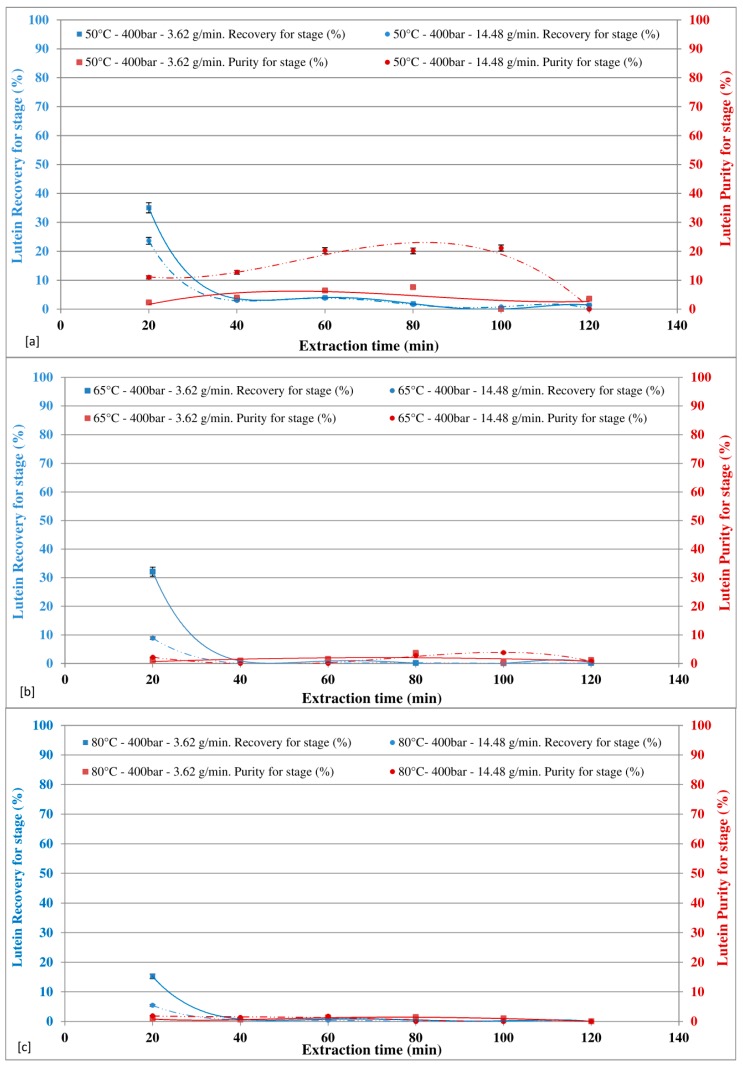
Effect of CO_2_ flow rate at different temperatures on lutein recovery and purity in extract at 400 bar.

**Figure 6:**
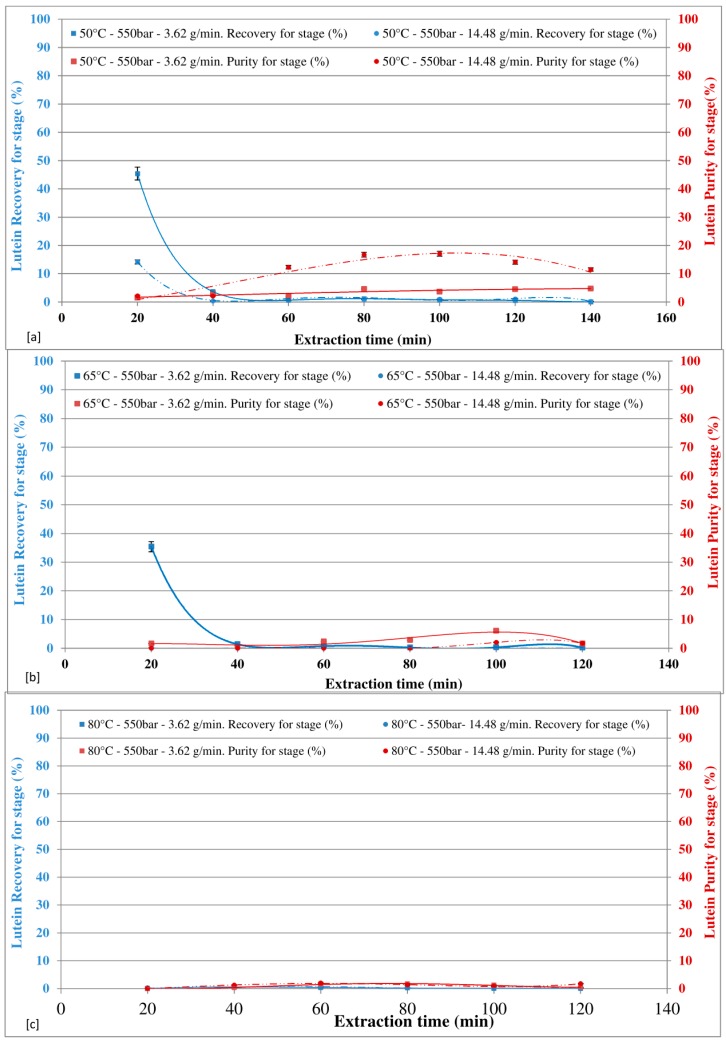
Effect of CO_2_ flow rate at different temperatures on lutein recovery and purity in extract at 550 bar.

The effects of CO_2_ flow rate (3.62 g/min and 14.48 g/min), as function of the extraction time, on SFE-CO_2_ lutein extraction were investigated by operating the temperature of the reaction chamber to 50 °C, 65 °C, and 80 °C at 400 bar (Figure 5a–c) and at 550 bar (Figure 6a–c).

At 400 bar (Figure 5a–c), for all the temperatures investigated, the lutein recovery was affected by the CO_2_ flow rate for the first extraction cycle (20 min), after which the effect of the CO_2_ flow rate was negligible. In particular, around 40% lutein recovery was achieved in first extraction cycle (20 min), with a CO_2_ flow rate of 3.62 g/min and about 25% at CO_2_ flow rate of 14.48 g/min. The lutein purity was affected by the CO_2_ flow rate at 50 °C, while at both 65 °C and 80 °C the effect of CO_2_ flow rate was negligible. The maximum purity of about 20% lutein was achieved at 50 °C with a CO_2_ flow rate of 14.48 g/min and an extraction time of 100 min.

At 550 bar (Figure 6a–c), the lutein recovery was affected by the CO_2_ flow rate at 50 °C for the first extraction cycle (20 min), while at both 65 °C and 80 °C the effect of CO_2_ flow rate was negligible for all the extraction stages. In particular, at 50 °C around 45% lutein recovery was achieved in first extraction cycle (20 min) with a CO_2_ flow rate of 3.62 g/min and about 15% at CO_2_ flow rate of 14.48 g/min. The lutein purity was affected by the CO_2_ flow rate at 50 °C and 65 °C, while at 80 °C the effect of CO_2_ flow rate was negligible. The maximum purity of about 20% lutein was achieved at 50 °C with a CO_2_ flow rate of 14.48 g/min and an extraction time of 100 min.

The higher lutein recovery achieved in first extraction cycle with respect to the astaxanthin recovery in the similar experimental condition could be explained by considering the higher driving force of lutein mass transfer between the inside and outside the algal cell as reported by several research groups [24,30,46].

By increasing temperature from 50 °C to 80 °C, both at 400 bar and at 550 bar, the recovery and purity decrease.

### 3.3. Effect of Temperature and Pressure on Global Recovery of Astaxanthin and Lutein

**Figure 7:**
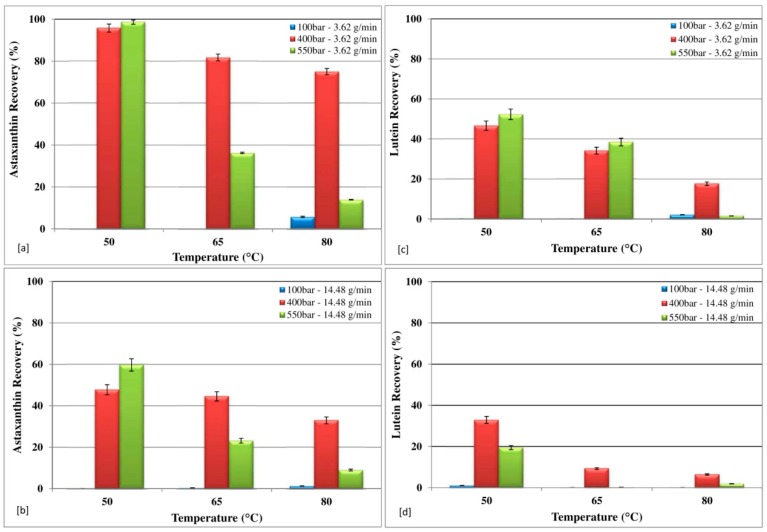
Effect of temperature and pressure on global recovery of astaxanthin and lutein.

The effect of temperature on astaxanthin total recovery, at the end of the extraction (120 min, sum of each stage), at different pressures with the CO_2_ flow rate of 3.62 g/min is shown in Figure 7a, while Figure 7b shows the effect of the CO_2_ flow rate of 14.48 g/min in the same conditions. By increasing temperature, a decrease in total astaxanthin recovery was observed. This finding may be explained by the possible increase of the thermal degradation rate of astaxanthin due to the increase of temperature. The opposite trend can be observed for the pressure, as the higher the pressure, the higher the astaxanthin extraction. It is evident that an increase in pressure increases the solubility of astaxanthin, and same trend was observed by Machmudah et al. [46]. In terms of CO_2_ flow rate, the lower the CO_2_ flow rate, the higher the astaxanthin recovery. The highest total recovery of astaxanthin, equal to 98.6%, was observed at 50 °C and 550 bar, with a CO_2_ flow rate of 3.62 g/min. With a CO_2_ flow rate of 3.62 g/min, increasing the extraction temperature to 65 °C and to 80 °C, the astaxanthin recovery drops to 36% and 14%, respectively. This effect may be explained considering the thermal degradation of subunits of astaxanthin as extensively reported in the literature [3,28,48,49]. These results show that relatively low temperature (50 °C) at 400 bars was optimum to effectively complete astaxanthin extraction.

The effect of temperature on lutein total recovery, at the end of extraction (120 min, sum of each stage), at different pressures with the CO_2_ flow rate of 3.62 g/min is shown in Figure 7c, while Figure 7d shows the effect of the CO_2_ flow rate of 14.48 g/min at the same conditions. The amount of lutein in the extract significantly decreased with increasing temperature, due to the thermal instability of carotenoids, as reported by several authors [50,51]. With a CO_2_ flow rate of 3.62 g/min, at 50 °C and 65 °C, the higher recovery was found at 550 bar; at a temperature of 80 °C, the higher recovery was found at 400 bar. With a CO_2_ flow rate of 14.48 g/min, for all the tested temperature, the higher recovery was found at 400 bar.

The highest total recovery of lutein, equal to 52%, was observed at 50 °C and 550 bar, with a CO_2_ flow rate of 3.62 g/min. With a CO_2_ flow rate of 3.62 g/min, increasing the extraction temperature to 65 °C and to 80 °C, the astaxanthin recovery drops to 35% and 14%, respectively. The lowest lutein recovery of 7% was achieved at 80 °C and 400 bar with a flow rate of 14.48 g/min.

### 3.4. Effect of Temperature and Pressure on Recovery of FAs

**Figure 8:**
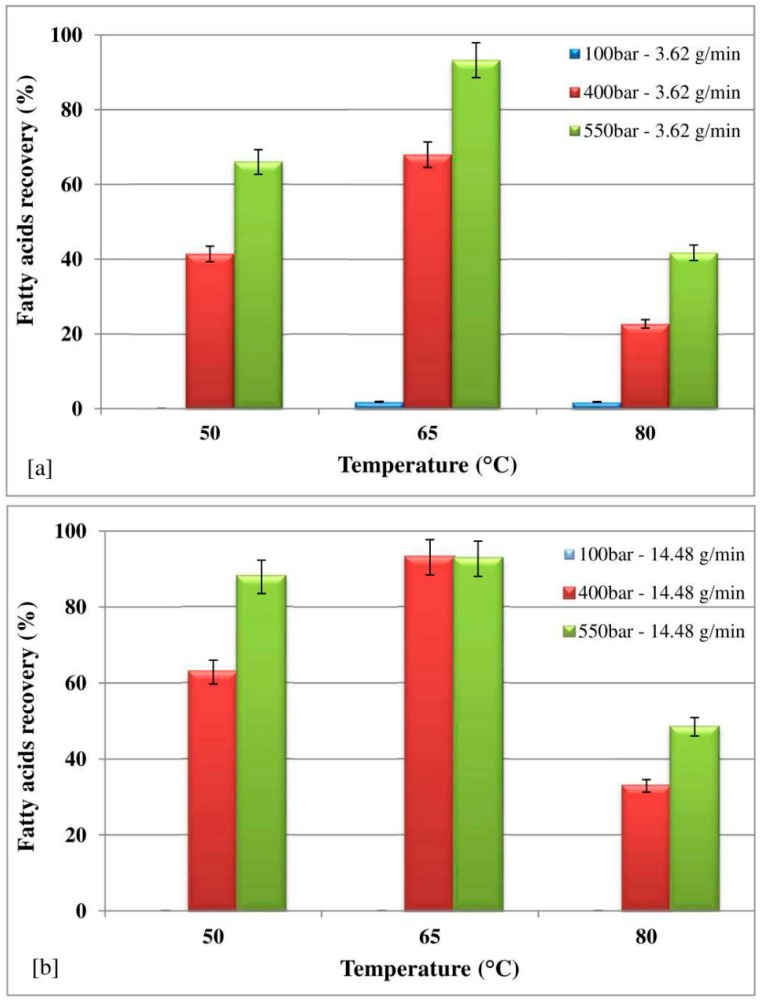
Effect of temperature and pressure on global recovery of fatty acids.

The recovery of FAs was investigated in two extracts, at 20 and 40 min, due to unavailability of FAs (lower than the detection limits) from the third extract (60 min), at different temperatures (50 °C, 65 °C and 80 °C) and pressures (100 bar, 400 bar and 550 bar) with both CO_2_ flow rates (3.62 g/min, Figure 8a; 14.48 g/min Figure 8b). As shown in Figure 8, the higher the pressure, the higher the FAs recovery with both the CO_2_ flow rates, for all the temperatures investigated, excluding the operative condition of a temperature of 65 °C and a CO_2_ flow rate of 14.48 g/min, with which the FAs recoveries at 400 bar and at 550 bar were comparable. This trend is probably due to two opposite effects as the pressure rises at a constant temperature: an enhancement in the density of CO_2_ in supercritical and a reduction CO_2_ diffusion coefficient can be observed [28,44]. In particular, increase in the density leads to an increase in its solvating power and thus enhancement of the extraction yield. On the other hand, a decrease in the diffusion coefficient of CO_2_ leads to a decrease in the ability of a fluid to penetrate the solid matrix, causing a reduction in the extraction yield. At the lowest temperature studied (50 °C), the dominant effect was the decrease in the diffusivity of CO_2_ when the pressure increased, while at the optimum temperature (65 °C), an enhancement was observed in the solvating. The highest temperature (80 °C) reduced fatty acid recovery.

Experimental findings also highlight that the higher the CO_2_ flow rate, the higher the FAs recovery; however, comparable FAs recoveries were achieved at 65 °C and 550 bar for both the CO_2_ flow rates investigated.

The characterization of the fatty acid (FA) extracted from *H. pluvialis* in red phase, including the effect of temperature and pressure on FAs classes at the CO_2_ flow rates of 3.62 and 14.48 g/min, respectively, are reported in Table 3, in which the theoretical content for each FAs species is also enclosed. Comparing the extracted amounts with the theoretical contents, it is possible to observe that the highest recoveries of SFAs, MUFAs, and PUFAs with a CO_2_ flow rate of 3.62 g/min were found at 65 °C and 550 bar, with values of about 86%, 90%, and 99%, respectively. With a CO_2_ flow rate of 14.48 g/min, the highest recoveries of SFAs and PUFAs, equal to 86% and 98%, were found at 65 °C and 400 bar, while the highest recovery of MUFAs, equal to 91%, was found at 65 °C and 550 bar.

### 3.5. Comparison of Astaxanthin, Lutein, and FAs Global Recovery at Different Operative Conditions

The comparison of the *H. pluvialis* extracts for total recovery of astaxanthin and lutein (120 min, sum of each extraction stage) and FAs (40 min, sum of first and second extraction stage) at different operative conditions shows that the FAs were a major component of the extract (Table 4). The maximum recoveries of astaxanthin (19.72 mg/g) and lutein (4.03 mg/g) were achieved with a CO_2_ flow rate of 3.62 g/min at 50 °C and 550 bar. The maximum recovery of FAs (21.41 mg/g) was achieved with a CO_2_ flow rate of 3.62 g/min at 65 °C and 550 bar.

The literature suggests that increasing temperature decreases the recovery of astaxanthin and lutein, while there is less thermal degradation of FAs with respect to astaxanthin and lutein [28,48,52,53,54].

## 4. Conclusions

In this work, SFE-CO_2_ extraction of astaxanthin, lutein, and FAs from *H. pluvialis* microalgae in red phase was investigated at different temperatures (50 °C, 65 °C, and 80 °C) and pressures (100 bar, 400 bar, and 550 bar) with CO_2_ flow rates of 3.62 and 14.48 g/min. Experimental findings show that the SFE-CO_2_ is more selective for the extraction of FAs with respect to astaxanthin and lutein. By using a single cell reactor, recoveries of astaxanthin, lutein, and FAs, which were equal to 98.62%, 52.32%, and 93.25%, were found at lower temperatures, and high pressure with the lower CO_2_ flow rate. Results highlighting the influence of extraction operative conditions on the maximum recovery of these “high-value, added compounds”. The maximum extractions of astaxanthin and lutein were achieved at 50 °C and 550 bar with a CO_2_ flow rate of 3.62 g/min. The maximum extraction of FAs was found at 65 °C and 550 bar, with a CO_2_ flow rate of 3.62 g/min. Among FAs species, PUFAs were extracted with the highest recovery.

Greater purities of astaxanthin and lutein were found when their recoveries were very low; therefore, this could represent a critical point for the development of this technology in the extraction of astaxanthin and lutein with respect to other carotenoids that have a similar polarity with CO_2_.

## Figures and Tables

**Table 1:** The experimental plan.

Operative Conditions			
Temperature (°C)	Pressure (bar)	CO_2_ Flow Rate (g/min)	Biomass Load (g)
50	100	3.62	1.38
50	100	14.48	1.36
50	400	3.62	1.37
50	400	14.48	1.38
50	550	3.62	1.38
50	550	14.48	1.36
65	100	3.62	1.34
65	100	14.48	1.34
65	400	3.62	1.33
65	400	14.48	1.32
65	550	3.62	1.35
65	550	14.48	1.34
80	100	3.62	1.35
80	100	14.48	1.34
80	400	3.62	1.31
80	400	14.48	1.38
80	550	3.62	1.34
80	550	14.48	1.34

**Note:** Biomass load is expressed on dry basis.

**Table 2:** Effect of temperature, pressure, and CO_2_ flow rate on total extraction yield.

Operative Conditions			Total Extraction Yield (mg/g)
Temperature (°C)	Pressure (bar)	CO_2_ Flow Rate (g/min)	
50	100	3.62	0.1
50	100	14.48	17.5
50	400	3.62	136.4
50	400	14.48	20.7
50	550	3.62	237.4
50	550	14.48	53.2
65	100	3.62	4.8
65	100	14.48	1.4
65	400	3.62	279.2
65	400	14.48	34.6
65	550	3.62	185.8
65	550	14.48	15.6
80	100	3.62	10.9
80	100	14.48	8.5
80	400	3.62	160.5
80	400	14.48	28.0
80	550	3.62	60.4
80	550	14.48	189.5

**Note:** Standard deviation was less than 5% in all operative conditions.

**Table 3:** Comparison of different operative conditions for fatty acid recovery at CO_2_ flow rates of 3.62 g/min and 14.48 g/min.

**Class of Fatty Acids (mg/g)**	**Operative Temperature (°C)**									**Theoretical Content**
	**50**			**65**			**80**			
	**Operative Pressure (bar) at CO_2_ Flow Rate of 3.62 g/min**									
	**100**	**400**	**550**	**100**	**400**	**550**	**100**	**400**	**550**	
SFAs	nd	0.64	5.5	0.42	4.8	5.57	0.41	0.38	0.47	6.45
MUFAs	nd	0.49	0.35	<Ldl	0.94	4.92	<Ldl	0.27	0.12	5.44
PUFAs	nd	8.38	9.3	<Ldl	9.87	10.92	<Ldl	4.57	8.98	11.06
	**Operative Pressure (bar) at CO_2_ Flow Rate of 14.48 g/min**									
	**100**	**400**	**550**	**100**	**400**	**550**	**100**	**400**	**550**	
SFAs	nd	0.58	3.97	nd	5.57	5.46	nd	0.41	0.49	6.45
MUFAs	nd	4.56	5.25	nd	4.9	4.97	nd	0.84	2.57	5.44
PUFAs	nd	9.28	10.97	nd	10.9	10.85	nd	6.32	8.08	11.06

**Note:** nd = not detected; <Ldl = lower than the detection limit; standard deviation was less than 5% at all operative conditions.

**Table 4:** The recovery of astaxanthin, lutein, and FAs at different operative conditions.

Recovery (mg/g_dry biomass_)	Temperature (°C)	CO_2_ Flow Rate of 3.62 g/min			CO_2_ Flow Rate of 14.48 g/min		
		Pressures (bar)					
		100	400	550	100	400	550
Astaxanthin	50	0.10	19.16	19.72	0.01	9.55	11.94
	65	0.06	16.34	7.24	0.07	8.91	4.62
	80	1.16	15.00	2.78	0.25	6.58	1.79
Lutein	50	0.08	3.60	4.03	0.08	2.53	1.50
	65	0.00	2.63	2.96	<Ldl	0.71	0.01
	80	0.16	1.36	0.12	<Ldl	0.49	0.15
FAs	50	nd	9.5	15.15	nd	14.43	20.19
	65	0.42	15.6	21.41	nd	21.37	21.29
	80	0.41	5.21	9.57	nd	7.57	11.14

**Note:** nd = not detected; <Ldl = lower than the detection limit; standard deviation was less than 5% at all operative conditions.
